# Prefrontal Activity and Connectivity with the Basal Ganglia during Performance of Complex Cognitive Tasks Is Associated with Apathy in Healthy Subjects

**DOI:** 10.1371/journal.pone.0165301

**Published:** 2016-10-31

**Authors:** Leonardo Fazio, Giancarlo Logroscino, Paolo Taurisano, Graziella Amico, Tiziana Quarto, Linda Antonella Antonucci, Maria Rosaria Barulli, Marina Mancini, Barbara Gelao, Laura Ferranti, Teresa Popolizio, Alessandro Bertolino, Giuseppe Blasi

**Affiliations:** 1 University of Bari ‘Aldo Moro’—Department of Basic Medical Science, Neuroscience, and Sense Organs, 70124, Bari, Italy; 2 University of Bari ‘Aldo Moro’—Department of Basic Medicine, Neuroscience, and Sense Organs, 70124 Bari, Italy and University of Bari ‘Aldo Moro’, “Pia Fondazione Cardinale G. Panico”—Department of Clinical Research in Neurology, 73039 Tricase, Lecce, Italy; 3 University of Perugia—Department of General and Experimental Medicine, 06123, Perugia, Italy; 4 IRCCS “Casa Sollievo della Sofferenza”, 71013, San Giovanni Rotondo, Italy; 5 Bari University Hospital—Psychiatry Unit, 70124, Bari, Italy; Universitair Medisch Centrum Groningen, NETHERLANDS

## Abstract

**Objective:**

Convergent evidence indicates that apathy affects cognitive behavior in different neurological and psychiatric conditions. Studies of clinical populations have also suggested the primary involvement of the prefrontal cortex and the basal ganglia in apathy. These brain regions are interconnected at both the structural and functional levels and are deeply involved in cognitive processes, such as working memory and attention. However, it is unclear how apathy modulates brain processing during cognition and whether such a modulation occurs in healthy young subjects. To address this issue, we investigated the link between apathy and prefrontal and basal ganglia function in healthy young individuals. We hypothesized that apathy may be related to sub-optimal activity and connectivity in these brain regions.

**Methods:**

Three hundred eleven healthy subjects completed an apathy assessment using the Starkstein’s Apathy Scale and underwent fMRI during working memory and attentional performance tasks. Using an ROI approach, we investigated the association of apathy with activity and connectivity in the DLPFC and the basal ganglia.

**Results:**

Apathy scores correlated positively with prefrontal activity and negatively with prefrontal-basal ganglia connectivity during both working memory and attention tasks. Furthermore, prefrontal activity was inversely related to attentional behavior.

**Conclusions:**

These results suggest that in healthy young subjects, apathy is a trait associated with inefficient cognitive-related prefrontal activity, i.e., it increases the need for prefrontal resources to process cognitive stimuli. Furthermore, apathy may alter the functional relationship between the prefrontal cortex and the basal ganglia during cognition.

## Introduction

Apathy is defined as reduced motivation towards goal-directed behavior, a flattened affect, emotional indifference and a restricted response to important life events [1–2]. It implies diminished motivation and effort to perform personal and social everyday activities [1, 3]. In addition to being associated with a broad range of brain disorders [4–7], apathy is also present in elderly and young healthy subjects [8–12].

The brain circuitry that sustains apathy has been investigated primarily in clinical and elderly populations. Many of these studies have demonstrated an association between apathy and structural anomalies, such as lesions of lateral or medial areas of the prefrontal cortex (PFC) [13–14] and decreased PFC volume [15–18]. Furthermore, functional imaging studies in dementia and in post-stroke patients corroborate the relationship between frontal alterations and apathy. In particular, previous reports have indicated associations between apathy and reduced glucose metabolism and frontal rCBF perfusion [19–20], as well as a negative correlation between rCBF perfusion and apathy scores [21]. Other studies also suggested a link between apathy and abnormalities in the basal ganglia (BG). In particular, this trait has been related to focal lesions of the globus pallidus, the caudate and the putamen [22–24], as well as to BG hypoperfusion [25–26] and reduced BG volume [27–28]. Moreover, apathy is a frequent symptom in clinical conditions that are characterized by BG impairment [4, 29–30]. A small number of studies have suggested the involvement of other brain regions, such as the cingulate cortex, the insula or the premotor cortex [31–32], in apathy, but these results require replication. Overall, the current evidence is consistent in suggesting a role for both the PFC and the BG in apathy in clinical and elderly populations. Accordingly, it has been hypothesized that apathy may be subtended by a dysfunction in the neuronal circuit that includes both BG and PFC [33–35], which are interconnected at both the structural and functional levels [36–38].

The loop between the BG and the dorsolateral prefrontal portion of the PFC (DLPFC) is deeply involved in cognitive processing, as indicated repeatedly by studies that focused on working memory and attentional processing [39–41]. Apathy has also been correlated with anomalies in cognitive functions, as revealed by findings in clinical populations; these findings highlighted a relationship between high levels of apathy and poorer performance on tests for working memory and attention [42–46]. Similarly, apathy has been associated with cognitive impairment in elderly individuals who are not affected by neurological or psychiatric conditions [9, 47–49].

Given that most of studies of apathy have been performed in elderly or clinical populations, it is important to investigate this trait in healthy young subjects, thus nullifying the possible impact of other illness-related variables or aging on brain physiology. To our knowledge, only one recent study has addressed the relationship between apathy and brain imaging phenotypes in healthy young individuals. In that study [32], the authors investigated behavioral apathy (i.e., a sub-domain of apathy that is characterized by a lack of physical engagement, productivity and initiative). The results of this study indicated that behavioral apathy is associated with greater recruitment of the supplemental motor area and the motor cingulate, as well as lower functional connectivity between these brain regions during a reward task. However, to our knowledge, no studies have been performed to investigate the relationship between apathy and brain correlates of high order cognitive processing, such as working memory and attention, in healthy young subjects.

Here, we aimed to investigate the association of apathy with brain activity and functional connectivity during working memory and attentional processing in healthy young individuals. Given our strong a priori hypothesis concerning the role of the basal ganglia and the PFC in apathy, we focused our analyses on those regions. Based on current knowledge, we hypothesized that greater levels of apathy would be associated with sub-optimal activity and connectivity in the DLPFC and the BG during these cognitive processes.

## Methods

### Subjects

Three hundred eleven healthy subjects (146 males, mean age + SD = 27.3 + 6.79 years) were included in the study. All subjects were evaluated using the Structured Clinical Interview [50] for the Diagnostic and Statistical Manual of Mental Disorders to exclude any actual or past psychiatric disorder. A standard MRI procedure was used to exclude brain structural alterations or illness. Other exclusion criteria were a history of drug or alcohol abuse, active drug use in the past year, head trauma with loss of consciousness and any significant medical condition. IQ (WAIS-R [51]), handedness (Edinburgh Inventory [52]), and socio-economic status (Hollingshead Four Factor Index, [53]) were also measured (Table 1). Handedness was determined by using the Edinburgh Inventory [52], which assesses the dominance of a person's right or left hand in everyday activities. The scores ranged from -1 (totally left handed) to 1 (totally right-handed). Socio-economic status was determined based on reports of paternal and maternal education and occupation by using the Hollingshead Four Factor Index [53]. This index ranges from 8 to 66 (8 = lowest level of occupational status and education). Furthermore, all subjects completed an apathy assessment and underwent one or more of the fMRI procedures described below.

**Table 1:** Demographic data and Apathy Scale (AS) data for the whole sample (All) and for the subsamples performing the working memory (N-Back) and attentional control (VAC) tasks.

	*Demographic Data*						*Apathy Scale *
	N		Age	Handedness	Socio-economic status	IQ	
**All**	311	***mean***	27.3	0.72	41.13	108.3	9.46
	146 ♂	***sd***	6.79	0.48	16.29	12.15	3.83
**N-Back **	247	***mean***	27.2	0.73	42.42	108.49	9.53
	119 ♂	***sd***	6.96	0.47	16.18	12.40	3.85
**VAC **	201	***mean***	27	0.72	42.42	109.645	9.16
	94 ♂	***sd***	5.4	0.48	16.7	11.81	3.25

The present study was approved by the local institutional review board, i.e. the “Independent Ethical Committee” at the University of Bari ‘Aldo Moro,’ and written informed consent was obtained from all subjects after a full explanation of all procedures was provided.

All the procedures were performed according to the Declaration of Helsinki. The data of this study are available at the 4TU.ResearchData repository; http://dx.doi.org/10.4121/uuid:d5ec1a8a-6c61-4f19-8af1-b728bb07e8c0.

### Apathy Scale

Apathy was measured in all individuals using the self-administered form of the Starkstein’s Apathy Scale (AS) [54], which is a reduced version of the Marin’s Apathy Evaluation Scale [55]. The AS was developed by removing redundant items from the Marin’s Apathy Evaluation Scale based on a factor analysis and included a total of 14 questions (e.g., “Do you have plans and goals for the future?”). Each item is rated on a four-point scale. The total score ranged from 0 to 42, with higher scores indicating greater apathy levels. The AS has adequate reliability, as well as good one-week test-retest and inter-rater reliability [54].

The Shapiro-Wilk normality test was used to assess the normality of the distribution for demographic characteristics and AS scores (p<0.05). Considering that the AS scores, age, handedness and socio-economic status were not normally distributed (see below), we used Spearman’s correlations and Kruskal-Wallis tests as needed to investigate putative relationships between demographic characteristics and AS scores. The statistical threshold was set at p<0.05.

### fMRI tasks

Two hundred forty-seven participants (119 males; mean age 27.20 + 6.96 years; Table 1) performed the N-Back task, which is a paradigm that has been used extensively to evaluate brain activity during working memory (WM) tasks [56–58]. ‘‘N-Back” refers to how far back in a sequence of stimuli the subject can recall. The stimuli consisted of numbers (1–4) shown in random sequence and displayed at the points of a diamond-shaped box. A non-memory-guided control condition (0-back) simply required the subjects to identify the stimulus currently seen. In the working memory condition, the task required the recollection of a number seen one (1-Back), two (2-Back) or three stimuli (3-Back) beforehand, while continuing to encode additional incoming stimuli. The stimuli were arranged in a block design, consisting of eight 30 sec blocks: four blocks of the control condition alternating with four blocks of each WM condition. Each run lasted 4 min 8 sec.

To extend our findings to another cognitive domain, 201 subjects (94 males; mean age 27 ± 5.4 years; Table 1) performed the Variable Attentional Control (VAC) task, which is a paradigm that has been used in several previous investigations [57, 59–63] and was designed to elicit increasing demands for attentional control processing. The stimuli were composed of arrows of three different sizes pointing either to the right or to the left; small arrows were embedded in medium-sized arrows, which were in turn embedded in a large arrow. The subjects were instructed by a cue word (big, medium or small) displayed above each stimulus to press a button corresponding to the direction of the large, medium or small arrows (right or left). To increase the level of attentional control required, the direction of the arrows was congruent or incongruent across all three sizes. This approach resulted in the following conditions: low level of attentional control (Low), for which all 3 sizes of arrows were congruent in direction, with the cue word BIG; intermediate level of attentional control (Int), for which two stimuli used, with the big arrow incongruent in direction to the small and the medium arrows in both stimuli and cue words of BIG in one stimulus and SMALL in the other stimulus; and high level of attentional control (High), for which two stimuli were used, with the medium-sized arrows incongruent in direction to the big and the small arrows in both stimuli and cue words of SMALL in one stimulus and MEDIUM in the other stimulus. A simple bold arrow pointing either to the left or right was used as a sensorimotor control condition.

The total number of stimuli was 241: 50 High (25 of each of the two stimuli that required a high degree of attentional control), 68 Int (34 of each of the two stimuli that required an intermediate degree of attentional control), 57 Low and 66 stimuli for the control condition. A fixation cross-hair was presented during the interstimulus interval, which ranged from 2 to 6 sec. The total duration of the task was 10 min 8 sec. The subjects were instructed to respond to task stimuli with the right hand using a button box (right button for ‘right’ response, left button for ‘left’ response) and to press the response button as rapidly and accurately as possible.

For both the N-Back and VAC tasks, the stimuli were presented via a back-projection system. The responses were recorded through a fiber optic response box, allowing measurement of the percent accuracy and the reaction time (in milliseconds). All subjects were trained to perform the task prior to the fMRI session. One hundred forty-one subjects performed both of the tasks used in this study.

### fMRI Data Acquisition

Blood oxygen level-dependent (BOLD) fMRI was performed on a GE Signa 3T scanner (GE Healthcare) equipped with a standard quadrature head coil. A gradient-echo planar imaging sequence (repetition time, 2000 ms; echo time, 30 ms; thickness, 4 mm; gap, 1 mm; flip angle, 90°; field of view, 24 cm; and matrix, 64 × 64) was used to acquire images while the subjects performed the tasks (N-back: 120 volumes for each run, 20 interleaved axial slices; VAC: 300 volumes, 26 interleaved axial slices). The first four scans were discarded to allow for T1 equilibration effects.

### fMRI

#### BOLD response

The analysis of the fMRI data was completed using Statistical Parametric Mapping 8 (SPM8; http://www.fil.ion.ucl.ac.uk/spm). Images of each subject were pre-processed and slice timing corrected using the centrally acquired slice as the reference slice. In particular, standard procedures for realignment to the mean image were performed using the Realign and Unwarp algorithm provided in SPM8 in order to compensate for non-linear signal distortions that may be induced by head motion. Furthermore, movement parameters were extracted to eventually exclude subjects with excessive head motion (>2 mm translation, > 2° rotation). The realigned images were resliced to a 2 mm isotropic voxel size, spatially normalized into a standard space (Montreal Neurological Institute template) with a 12 parameter affine model and smoothed using a 6 mm full-width half-maximum isotropic Gaussian kernel to minimize noise and to account for residual inter-subject differences.

In the N-Back first-level analysis, a box car model convolved with the hemodynamic response function at each voxel was used. Linear contrasts were then computed, producing a t statistical map for the 1-, 2- and 3-Back conditions, assuming the 0-Back condition as a baseline. Thus, a multiple regression analysis of the N-Back-related brain activity was then performed at the group level, using apathy as the continuous predictor, WM load (1-, 2-, and 3-Back) as the repeated-measures factor and age and gender as covariates of no interest (see below).

In the VAC task, the fMRI responses were modeled using a canonical hemodynamic response function. Vectors were created for each condition using the timing of correct responses. Using a t statistic, linear contrasts were computed for the three levels of attentional control (High, Int, and Low). Thus, a multiple regression analysis was performed on VAC-related brain activity, using apathy as the continuous predictor, attentional control load (High, Int, and Low) as the repeated-measures factor and age and gender as covariates of no interest. Because of our strong a priori hypothesis about the involvement of the DLPFC and of BG in working memory and attentional control processing [60, 64–66], as well as the putative involvement of these regions in apathy [34, 67], we used a statistical threshold of p<0.05, family-wise error (FWE) small-volume corrected within a region of interest that included DLPFC Brodmann’s areas 9, 10 and 46, as well as the BG, as defined by the WFU_PickAtlas [68].

To explore the association of brain activity with behavior, BOLD parameter estimates were extracted for both the N-back and VAC from the cluster that showed a significant association with apathy using MarsBaR (http://marsbar.sourceforge.net/) (see below).

#### Psychophysiological interactions (PPI)

A psychophysiological interaction (PPI) analysis [69] was performed to evaluate the association of apathy scores with DLPFC functional connectivity. For both the N-Back and VAC tasks, 5 mm ROIs centered on the clusters whose activity was associated with apathy (see results) were used as seed regions. PPI was calculated using the first eigenvariate of individual raw activation time courses, which were extracted by using a singular value decomposition method from a volume of interest (VOI) centered on the subject-specific peak cluster within the seed regions. These time courses were then mean-centered, high-pass filtered and deconvolved. A general linear model was computed using three regressors: a physiological regressor (the time course response in the VOI), a psychological regressor (task design) and a PPI term, which was calculated as the cross-product of the previous two terms. Thus, subject-specific statistical PPI contrast images were entered in second-level random effects multiple regressions, using apathy as the continuous predictor, task load as the repeated-measures factor and age and gender as covariates of no interest. Based on the relevance of the DLPFC/BG functional loop for both apathy and cognitive processing [34], we focused our investigation on the BG, which included the caudate, the putamen and the globus pallidus, as identified using the WFU Pickatlas. A statistical threshold of p<0.05, family-wise error (FWE) small-volume corrected within this ROI, was used for these analyses.

To further explore the association of brain connectivity with behavior, PPI values were extracted for both the N-back and VAC tasks from the cluster that showed a significant association with apathy using MarsBaR (http://marsbar.sourceforge.net/) (see below).

#### Correlation analysis

To explore the relationship between apathy and cognitive behavior, we calculated a parametric cognitive efficiency (PCE) score for both the N-Back and the VAC tasks, which takes into account the increase in cognitive load that is elicited by the working memory and attentional control tasks. In particular, we obtained an efficiency index as the ratio between the percent accuracy and the reaction time for each of the three loads of the tasks (1-, 2- and 3-Back for the N-back and Low, Int and High for the VAC). Then, we ranked each cognitive load (1-Back = 1; 2-Back = 2; 3-Back = 3 for the N-back; Low = 1; Int = 2; High = 3 for the VAC) and multiplied the efficiency index and the rank of each load. Thus, we summed the scores obtained for each cognitive load in order to obtain an individual PCE for each task. This procedure is simplified by the following formulas:

N-BackPCE=[(percentaccuracy1-Back/reactiontime1-Back)*1]+[(percentaccuracy2-Back/reactiontime2-Back)*2]+[(percentaccuracy3-Back/reactiontime3-Back)*3]

VACPCE=[(percentaccuracyLow/reactiontimeLow)*1]+[(percentaccuracyInt/reactiontimeInt)*2]+[(percentaccuracyHigh/reactiontimeHigh)*3]

The Shapiro-Wilk normality test was used to assess the normality of the distribution of the PCE scores (p<0.05). Considering that the N-Back PCE score was not distributed normally (see below), for each task, we performed separate Spearman’s correlation analyses between the PCE score and the BOLD parameter estimates or the PPI values extracted from significant clusters obtained in the activity and connectivity analyses. A statistical threshold of p<0.05 was used for these analyses.

To assess the relationship between PCE and AS scores, we used robust regression models, which allowed us to control for age and gender (see below) without being affected by violations of parametric assumptions. The statistical threshold was set at p<0.05.

## Results

### Apathy scores and Demographics

The Shapiro-Wilk normality test indicated that the AS scores, age, handedness and socio-economic status were not distributed normally (AS score W = 0.95, p<0.001; Age W = 0.83, p<0.001; handedness W = 0.97, p<0.001; socio-economic status W = 0.59, p<0.001). The AS scores (range = 2 to 26, mean ± SD = 9.46 ± 3.83; N-Back sample = 9.53 ± 3.85; VAC sample = 9.16 + 3.25) were greater in males than in females (Kruskal-Wallis Chi-squared = 7.29; p = 0.006) and correlated with age (Spearman’s rho = -0.18; p = 0.002) but not with handedness, socio-economic status and IQ (all p>0.05). Age and gender were thus used as covariates in all fMRI and behavioral analyses that included apathy.

### fMRI

#### Regional activity

**Fig 1:**
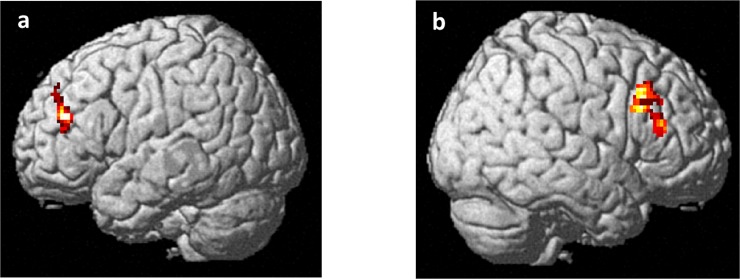
Rendered image of the brain depicting the dorsolateral prefrontal clusters whose activity correlated positively with apathy scores during (a) working memory and (b) attentional control tasks. See the text for statistics.

Analysis of the N-back data indicated a positive correlation between apathy scores and left middle frontal gyrus BOLD responses during the N-back task (BA 46; x -42, y 40, z 24; Z = 3.98; k = 145; p = 0.03 FWE-corrected) (Fig 1A). No interaction between apathy and working memory load was found. Similarly, analysis of the VAC data indicated a positive correlation between apathy scores and right middle frontal gyrus activity (BA 46; x 42, y 30, z 18; Z = 3.83; k = 228; p = 0.047 FWE-corrected) (Fig 1B). Again, no interaction was observed between apathy scores and attentional load.

#### PPI

**Fig 2:**
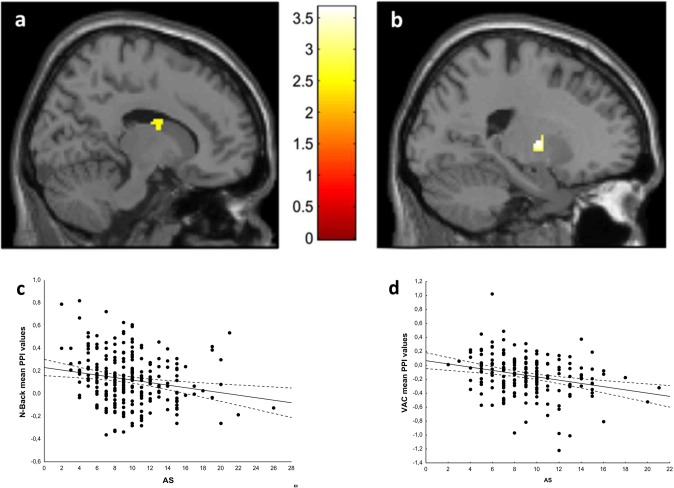
Sections of the brain depicting the basal ganglia clusters whose functional connections with the dorsolateral prefrontal cortex correlated negatively with apathy scores during (a) the working memory task and (b) the attentional control task. Scatterplots depicting the negative association between PPI values and apathy during (c) the working memory task and (d) the attentional control task. See the text for statistics.

A PPI analysis was performed using the DLPFC clusters associated with apathy scores during N-Back and VAC performance as seeds. A PPI analysis of the N-Back data revealed that apathy scores correlated negatively with functional connectivity between the DLPFC and the right BG (x18,y -2,z 22; Z = 3.71; k = 68; p = 0.036 FWE-corrected) (Fig 2A and 2C). No interaction between working memory load and apathy scores was present. Similarly, a PPI analysis of the VAC data revealed a negative correlation between apathy scores and functional connectivity between the DLPFC and the left BG (x -20, y -6, z 4; Z = 3.64; k = 70; p = 0.044 FWE-corrected) (Fig 2B and 2D). No interaction was observed between attentional load and apathy scores.

#### Correlation analysis

**Fig 3:**
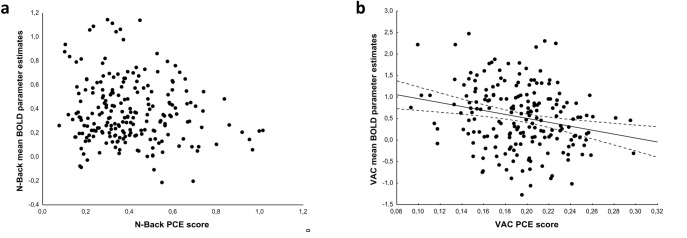
Scatterplots of Spearman’s test on cognitive behavior as indexed by a parametric cognitive efficiency score (PCE) and BOLD parameter estimates extracted from the dorsolateral prefrontal region associated with apathy, depicting (a) absence of correlation during working memory task and (b) negative correlation during attentional control. See text for statistics.

Behavioral data concerning the subjects who performed the N-Back task and the VAC task are reported in Table 2. The Shapiro-Wilk normality test indicated that the N-Back PCE score was not distributed normally (N-back PCE W = 0.95, p<0.001). Spearman’s correlations indicated a negative correlation between PCE scores and BOLD activity during the VAC task (Spearman’s Rho = -0.22; p = 0.002) (Fig 3B) but not during the N-back task (Spearman’s Rho = -0.08; p = 0.2) (Fig 3A). Spearman’s correlations between PPI and behavioral data revealed no significant results (p>0.05). Robust regression models did not indicate a relationship between apathy and PCE scores during the N-back task or the VAC task (all p> 0.05).

**Table 2:** Behavioral data for the subsamples performing the working memory (N-Back) and attentional control (VAC) tasks.

		N-BACK			VAC		
		1 Back	2 Back	3 Back	Low	Medium	High
**% Accuracy**	***mean***	95.5	85.3	77.9	99	89.9	83.5
	***sd***	8.1	14.7	16.2	4.3	9.5	13.7
**Reaction Time**	***mean***	527.9	536.2	492.9	781.9	931.2	1059.5
	***sd***	218.8	225.7	238.9	175	183.7	206.7
**PCE**	***mean***	0.40			0.19		
	***sd***	0.18			0.03		

## Discussion

Here we investigated whether apathy is associated with DLPFC and BG activity and functional connectivity during cognitive processing in healthy individuals. We found a positive correlation between apathy scores and prefrontal responses during working memory tasks, such that subjects with greater apathy also had greater DLPFC activity during this cognitive skill. Notably, we found a similar relationship when investigating the domain of attentional control processing. Moreover, greater levels of apathy were also associated with lower functional connectivity between the DLPFC and the BG during both cognitive processes. These results suggest a relationship between patterns of brain cognitive processing and apathy in healthy subjects.

The observed association between dorsolateral prefrontal activity and AS scores in healthy subjects is consistent with previous results obtained in clinical populations, suggesting that apathy may be subtended by damage that occurs primarily in the lateral prefrontal cortex [13]. The DLPFC is a key region for working memory and attention processing [70–72], and previous models suggested that for between-group comparisons, greater DLPFC activity despite worse or unaffected behavior, may be an index of inefficient prefrontal processing during cognition [58, 60, 70, 73]. In light of this model, our results that demonstrate a positive correlation between AS and DLPFC activity suggest that greater apathy in healthy subjects is linked with less efficient prefrontal processing of working memory and attentional stimuli. Consistent with this finding, our correlation analysis indicates that greater DLPFC activity linked with greater apathy predicts poorer behavioral performance in the attentional control task used in our study. Indeed, we found no significant correlation between the PCE score and DLPFC activity during working memory tasks. A possible explanation for this lack of a relationship is that the region that we found to be associated with apathy during working memory is less modulated by task load but is relevant for task execution [74]. Overall, our findings in healthy subjects strengthen previous evidence of an association between apathy and impaired cognition [42–45, 75–78], further suggesting a biological basis for this phenomenon and uncoupling this relationship from the effects of confounding factors related to disease or aging. Moreover, our findings highlight that the role of cognitive processing in apathy flanks those of emotion processing and mood [79–80].

Another finding of the present study is the relationship between apathy and DLPFC-BG connectivity during both working memory and attentional control tasks. In particular, we found that greater levels of apathy are associated with a weaker functional connection between inefficient clusters of prefrontal activity during cognitive processing and BG. These results are consistent with previous models that posited a role for reduced DLPFC-BG connectivity in apathy [33–35, 67] and with previous findings that indicated that bilateral lesions of the BG are associated with a severe form of apathy (auto-activation deficit) that is characterized by a complete loss of self-initiated goal-directed behavior [81]. The DLPFC-BG loop plays a crucial role in high-order cognitive processes, such as working memory and attentional control [64],and participates in a so-called “associative pathway,” in which information that arises from several associative areas is transmitted to the caudate and the anterior putamen and subsequently reaches the DLPFC through the thalamus [66, 82]. This pathway is involved in several cognitive processes and is crucial for the generation of context-dependent and goal-directed patterns of behavior [66, 82–83]. Indeed, previous models postulated that a neurobiological mechanism subtending apathy may imply a failure of the BG to engage the DLPFC, thus lowering the ability of the DLPFC to support goal-directed cognitive processing [35, 67].

In our PPI analysis, we found that AS scores for both tasks are correlated with the functional connection between the DLPFC and the contralateral BG. Previous findings indicated the existence of a bilateral interconnection between the DLPFC and the BG [84–85]. Furthermore, by lowering the statistical threshold of our analysis to an uncorrected threshold of p<0.005, we found that AS scores in the N-Back and VAC tasks also correlated with the functional connections between the DLPFC and the homolateral BG. Thus, it is possible that our PPI finding at the corrected p value is related to the statistical threshold used.

A potential limitation of the present study is that apathy is considered to be an aspect of depression, and correlations between apathy and depression scales have been reported [2]. Even if we excluded from the study all of the subjects with a current or past diagnosis of depression, as well as individuals with first-degree relatives affected by a psychiatric disorder, it remains possible that subclinical levels of depression might have affected our results. However, previous studies have suggested good discriminability between apathy and depression scores in both clinical [86–87] and non-clinical populations [11–12], as well as an adequate discriminant validity between apathy scales and depression scales [88]. Thus, it is possible that our results are not strongly impacted by subclinical levels of depression. Further studies should address this issue.

In conclusion, we provided evidence for a relationship between inefficient brain processing during cognition and apathy in healthy subjects in the absence of confounding factors, such as pathophysiological conditions or pharmacological treatment. These findings shed new light on our understanding of the link between apathy and brain processes that may be relevant to neurological and psychiatric conditions for which apathy is a central feature.
